# Low-carbohydrate diet in the treatment of type 2 diabetes mellitus (LoCaT): study protocol for a multicenter, randomized controlled trial

**DOI:** 10.1186/s13063-023-07427-5

**Published:** 2023-06-19

**Authors:** Xinyi Xia, Miao Xu, Yunjie Gu, Yangxue Li, Li Li, Jun Yin

**Affiliations:** 1 Department of Endocrinology and Metabolism, Shanghai Sixth People’s Hospital Affiliated to Shanghai Jiao Tong University School of Medicine, Shanghai Clinical Center for Diabetes, Shanghai Diabetes Institute, Shanghai Key Laboratory of Diabetes Mellitus, 600 Yishan Rd, Shanghai, 200233 China; 2 Department of Endocrinology and Metabolism, Ningbo First Hospital, 59 Liuting Street, Ningbo, 315000 Zhejiang Province China; 3 Department of Endocrinology and Metabolism, Shanghai Eighth People’s Hospital, 8 Caobao Road, Shanghai, 200235 China

**Keywords:** Type 2 diabetes mellitus, Randomized controlled trial, Study protocol, Low-carbohydrate diet, Canagliflozin

## Abstract

**Background:**

Low-carbohydrate diet (LCD) is an emerging therapy for type 2 diabetes mellitus (T2DM). Although its effect on glucose control has been confirmed in previous clinical trials, most of those studies have focused on comparing calorie-restricted LCD to iso-caloric low-fat diets. In this study, we aim to compare the effects of LCD and canagliflozin, a sodium-glucose cotransporter 2 inhibitor, in patients with T2DM.

**Methods:**

This is a multicenter, randomized controlled trial. We will recruit 120 patients with poor-controlled T2DM. Participants will be randomly divided into canagliflozin and LCD groups in a 1:1 ratio. The primary outcome is the change in hemoglobin A1C levels after the 3-month intervention. The secondary outcomes are the time in range and cost of antihyperglycemic agents. Exploratory outcomes include physical examination, body composition, glucose variability, appetite, glycolipid metabolism, liver lipid content, and urine glucose threshold.

**Discussion:**

No previous study has compared an LCD with antihyperglycemic agents. In LoCaT, participants’ metabolism will be assessed from multiple perspectives. It is believed that the finding obtained from this trial will optimize the treatments for patients with T2DM.

**Trial registration:**

Chinese Clinical Trial Registry ChiCTR1900027592. Registered on November 20, 2019.

**Supplementary Information:**

The online version contains supplementary material available at 10.1186/s13063-023-07427-5.

## Background

Diabetes mellitus (DM) is one of the most common metabolic disorders worldwide, and its prevalence is increasing annually. In 2019, 463 million adults (20–79 years) were estimated to have diabetes [1]. The prevention and treatment of diabetes have become a growing public health challenge. The current management of diabetes includes lifestyle interventions, medications, and bariatric surgery. Lifestyle interventions mainly include diet modification and exercise, and the low-carbohydrate diet (LCD) is currently one of the most popular dietary interventions for diabetes.

LCD requires carbohydrate intake lower than 26% of total daily energy intake without limitations on protein and fat intake. The antihyperglycemic efficacy of LCD reported in different studies varies significantly. For example, a study involving 400 people found that 10-week LCD (carbohydrate intake < 30 g/day) reduced fasting blood glucose by 1.9 mmol/L and glycated hemoglobin A1C (HbA1c) by 1%, 87% of the patients reduced or stopped insulin therapy, and the dose of oral antihyperglycemic agents was also reduced to different degrees [2]. In addition, a meta-analysis showed that LCD (carbohydrate < 26% of total energy intake) decreased HbA1c by 0.47%, 0.36%, and 0.17% at 3 months, 6 months, and a year, respectively, while moderate carbohydrate diets (26–45% of total energy intake) reduced HbA1c by only 0.06%, 0.06%, and 0.08% at 3 months, 6 months, and a year, respectively [3]. Notably, we observed significantly better efficacy of LCD in improving blood glucose levels in clinical practice than previously reported. However, previous clinical trials have often focused on comparing LCD to low-fat diets rather than blank controls or positive controls. Therefore, we designed this trial to determine the effect of LCD in patients with type 2 diabetes mellitus (T2DM) by comparing it with a positive control drug.

In the past few years, sodium-glucose cotransporter 2 inhibitors have become a new class of antihyperglycemic agents. They reduce the reabsorption of glucose in the kidney, excrete excess glucose to urine, and lower plasma glucose levels. In addition, large clinical trials have also shown that sodium-glucose cotransporter 2 inhibitors can increase plasma ketones and reduce body weight [4]. Notably, both LCD and sodium-glucose cotransporter 2 inhibitors have ketogenic effects. Thus, in this trial, canagliflozin, a sodium-glucose cotransporter 2 inhibitor, will be used as a parallel control for LCD, and its effects on blood glucose levels and metabolism will be compared.

Metabolic syndrome is a cluster of abdominal obesity, insulin resistance, hypertension, and hyperlipidemia [5], and it is common in patients with T2DM. Therefore, this trial will also compare the efficacy of the two interventions in weight loss, insulin sensitivity improvement, blood pressure reduction, islet β-cell improvement, and dyslipidemia management.

The main purpose of this trial is to compare the antihyperglycemic efficacy of LCD and canagliflozin. In addition, blood glucose fluctuations will be evaluated by continuous glucose monitoring and pharmacoeconomics. Moreover, the trial will also explore the effects of the two interventions on body fat, blood pressure, liver and kidney function, appetite, and urine protein and their clinical safety profiles. This trial innovatively adopts canagliflozin as the positive control for LCD, and it will provide solid evidence for the application of LCD in patients with diabetes mellitus.

## Methods/design

### Design

LoCaT is a multicenter, randomized, positive-controlled, parallel-group, and non-inferiority trial. Participants will be recruited from three academic hospitals in China: Shanghai Sixth People’s Hospital Affiliated to Shanghai Jiao tong University School of Medicine, Shanghai Eighth People's Hospital, and Ningbo First Hospital.

A total of 120 participants will be enrolled in the study. Competitive enrollment was used to recruit participants, with an allocation ratio of 1:1 for the canagliflozin and LCD groups. The participants will receive the intervention, and data will be collected. The report will be written according to Standard Protocol Items: Recommendations for Interventional Trials [6].

### Objectives

The primary objective of LoCaT is to evaluate whether LCD has a similar effect as canagliflozin on glucose control based on the change of HbA1c from baseline to the end. The secondary objectives are glucose variability and expense change of antihyperglycemic agents after a 3-month intervention.

### Participants

#### Inclusion criteria

- Voluntary participation and signed informed consent
- 18–70 years of age
- Diagnosis of T2DM according to the 2019 American Diabetes Association standards [7]
- Glycated hemoglobin ≥ 7.5% and ≤ 11%
- Willing to accept canagliflozin or LCD
- Plasma β-hydroxybutyric acid ≤ 0.3 mmol/L or negative urine ketone

#### Exclusion criteria

- Diabetic ketoacidosis-related causes such as a history of myocardial infarction, chronic pancreatitis, stomach cramps, or alcohol abuse (drinking more than three standard cups per day or at least one time drinking more than five standard cups per week)
- History of other metabolic diseases (such as thyroid dysfunction and Cushing’s syndrome)
- Type 1 diabetes mellitus
- The average proportion of carbohydrates in the diet < 40% in the past 6 months
- Those with fatty acid transport and oxidative disorders, such as carnitine deficiency and porphyria
- History of symptomatic cholelithiasis (except for cholecystectomy)
- History of lower extremity infection, gangrene, and foot ulcers
- Alanine transaminase and/or aspartate aminotransferase > 3 times the upper limit of normal or patients with active liver diseases
- Estimated glomerular filtration rate ≤ 60 mL/(min·1.73 m.^2^)
- Patients with urinary tract infections
- Malignant tumors occurring within 2 years
- Pregnant or lactating women
- Those who are participating in or have participated in other clinical studies within 4 weeks before the start of the study
- The researcher believes that the participant has a disease that affects the outcome assessment or is unsuitable for enrollment.

### Sample size

This is a non-inferiority trial, and the primary outcome is the change in HbA1c from baseline (△HbA1c) to 13 weeks after treatment. According to previous studies and our pilot study, the standard deviation of △HbA1c was estimated as 0.73 for both groups [8–10]. Sample size was calculated using PASS software (version 11.0), with an alpha of 0.025 and beta of 0.2, and 54 samples per group were needed. Considering a 10% loss during follow-up, a total of 120 samples is reasonable for this study.

### Recruitment, screening, and randomization

T2DM patients with poor blood glucose control will be recruited at three hospitals in Shanghai and Ningbo, China. First, clinical endocrinologists will introduce the purpose, risks, and benefits of the study to T2DM patients. Then, researchers will communicate with those interested in the trial and confirm whether they meet the inclusion and exclusion criteria. Finally, informed consent will be obtained, and the initial visit will start.

The table of random numbers will be generated by a statistician with particular random seeds. Variable block sizes of 4 and 6 will be used and stratified by whether HbA1c is between 7.5% and 8.5% or above 8.5% and whether the patients have taken antihyperglycemic agents in the past 3 months. Sealed envelopes containing group information will be prepared. The sequence number for each stratification will be typed on the surface of the envelope. The researchers will open the envelopes according to the sequence of enrollment and identify the group. Participants will be randomly assigned in a 1:1 ratio to the canagliflozin or LCD group. Each participant will have a unique study number.

### Intervention

All participants will be instructed to maintain their extant therapy and daily exercise schedules, and they will be advised to drink sufficient water every day (2–3 L).

#### Positive control: canagliflozin group

Participants randomized into this group will receive canagliflozin for 3 months, which will be taken before breakfast (100 mg/day). Meanwhile, they will be asked to maintain their extant dietary habits.

#### LCD group

Participants randomized into this group will be required to reduce their intake of carbohydrates to less than 20% per day. No restriction will be required for the total caloric intake or the type and quantity of fat and protein. They will receive general educational materials on dietary advice and individual one-on-one consultations to meet their needs. Every participant will be asked to report their daily meals for the first 10 days after the intervention to obtain personalized instructions.

### Outcome measurements

#### Primary outcome

The change in HbA1c level is the primary outcome of this trial. It is defined as HbA1c after 3 months of treatment minus HbA1c at baseline.

#### Secondary outcomes

The secondary outcomes of this trial are the time in range and cost of antihyperglycemic agents. The time in range is defined as the proportion of time in the euglycemic range when using the FreeStyle Libre H Sensor (Abbott, England). Medications, including schedules and doses, at baseline and changes over 3 months will be recorded. The reduction or withdrawal of antihyperglycemic agents will be directed by an endocrinologist. The costs of the agents will be also recorded.

#### Exploratory outcomes

Physical examination and body composition test: heart rate, blood pressure, body mass index, and the waist-to-hip ratio will be recorded at each visit. Furthermore, body composition will be examined via bioelectrical impedance analysis (MC-780MA, Tanita, Japan).

Continuous glucose monitoring: The FreeStyle Libre H Sensor will be inserted by the same specialized operator at each center. The FreeStyle Libre H Sensor, accompanied by a reader, can provide glucose monitoring every 15 min for 14 days. Three days later, after the initial visit, participants in the LCD group will receive a FreeStyle Libre H Reader so that they can monitor their glucose levels and adjust their diet by themselves. They will be requested to provide their 24-h glucose levels to the researcher to obtain personalized diet instructions. The canagliflozin group will not get the reader and will be blinded to the monitoring results to avoid adjusting the meal or other behaviors, which may interfere with the results of the study. After a 3-month intervention, all participants will receive the sensor again, and they will be blinded to the monitor results to reduce interference with the final results.

Diet investigation and appetite scale: The dietary intake and appetite scale will be recorded at the initial visit and every month. Appetite, including hunger, fullness, and desire to eat, will be assessed using the 100 mm visual analog scale, with 0 (left) and 100 (right) representing the least and the most, respectively.

Blood test: At baseline, participants will eat a standard breakfast (542 kcal energy, 55% carbohydrate, 38% fat, and 7% protein) and undergo blood tests within 4 h. The specific items and time schedules of blood tests are listed in Table 1. At the final visit, participants in the canagliflozin group will consume a standard meal, and those in the LCD group will have an iso-caloric LCD meal (567 kcal energy, 10% carbohydrate, 79% fat, and 11% protein). Participants in both groups will repeat the tests performed at the initial visit. The LCD group will receive an additional visit with a standard meal to assess the urine glucose threshold. In addition, fasting glucose, insulin, C-peptide, β-hydroxybutyric acid, and 2-h postprandial glucose will be assessed at visits 1 and 2.

**Table 1:** Items and schedule of blood tests

	Time(min)					
	0	30	60	120	180	240
Plasma glucose, insulin, and C-peptide	XX	XX	XX	XX	XX	XX
Free fatty acid and triglyceride	XX			XX		XX
β-Hydroxybutyric acid	XX			XX		XX
HDL-C, LDL-C, TC, apo A1, apo B, and apo E	X					
sd-LDL	X					
Electrolytes	X					
Renin–angiotensin–aldosterone system	X					

XX, baseline and final visits in the two groups and additional visits in the LCD group; X, baseline and final visits in the two groups

Ultrasound: At the initial and final visits, FibroScan and heart ultrasound will be performed to assess the degree of fatty liver disease and heart function.

Urine test: At the initial and final visits, the urinary albumin-creatinine ratio will be measured. Additionally, urine glucose, sodium, and potassium levels will be examined at 2 and 4 h after breakfast at these two visits. The renal threshold for glucose will be calculated according to previously published protocols [11, 12].

Safety: Hypoglycemia, ketoacidosis, and other adverse events will be recorded during the trial. Records include, but do not limit to, the relationship with the interventions, actions taken, and outcomes.

### Data collection and confidentiality

Researchers at each center will collect data according to the timeline schedule shown in Table 2. Researchers from each center will receive their own account to upload data and will be blinded to other centers.

**Table 2:**
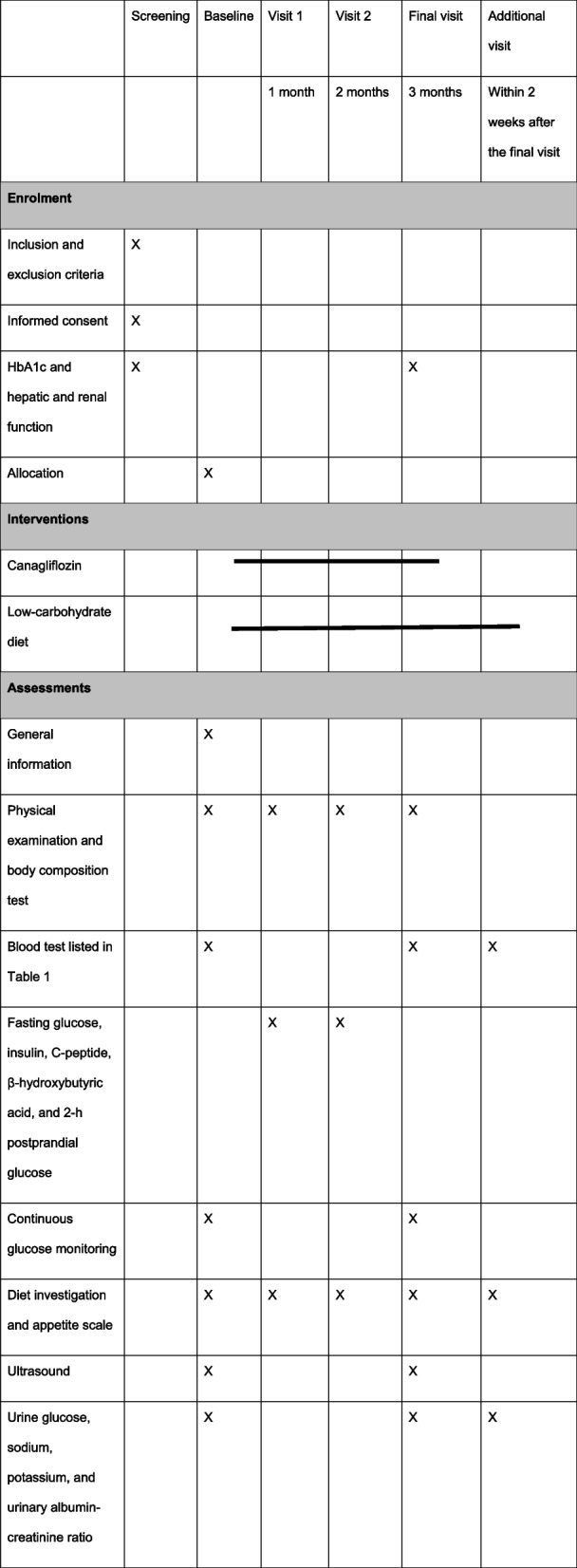
Participant schedule

During this trial, the privacy of all participants will be protected. Study documents, study reports, publications, and any other publicly published information will not include the participants’ names or other private information except as required by law. The collection, transmission, processing, and storage of participants’ information will comply with relevant laws and regulations to protect personal data from disclosure. Confidentiality of participants’ information will be strictly enforced by researchers. Confidentiality also covers information related to biological samples, in addition to the participants’ clinical information.

### Compliance and safety

For the canagliflozin group, the compliance of participants will be judged based on whether they take the drug according to the instructions and whether they add new treatment by themselves. For the LCD group, participants will be required to report the data of the FreeStyle Libre H Reader every day at the start of the intervention to ensure compliance with the diet. Furthermore, researchers will ask participants in the LCD group to report their whole-day diet randomly, about 4–5 times per month, and those in the canagliflozin group to report monthly.

All the adverse events occurring during the trial will be recorded. The records should include, but are not limited to, the time, signs, laboratory results, treatments, and outcomes. The relationship with adverse events will be determined by a clinician. Participants will be educated on how to handle emergency situations. For example, they need to have fast-acting carbohydrates when they have hypoglycemic symptoms and/or blood glucose levels lower than 3.9 mmol/L and report the situation to the researchers to get advice on adjusting their antihyperglycemic agents. The dose and frequency of canagliflozin are not allowed to be adjusted by the participants. If they do not tolerate or have adverse reactions, the researchers will be informed and decide the action according to the participant’s situation, such as drug interruption or withdrawal. When hypoglycemia occurs, the reduction order of antihyperglycemic agents is suggested to be from insulin injection and insulin secretagogues to other drugs.

### Data management and quality control

The Clinical Research Center of Shanghai Sixth People’s Hospital Affiliated to Shanghai Jiao Tong University School of Medicine (listed as Clinical Research Center below) will participate in center coordination, data audition, and the decision of study suspension and withdrawal due to serious adverse events. All data collected from participants will be entered into a database via a clinical trial management public platform (ResMan). The primary investigators of each center and statisticians have access to the final trial dataset. The study schedule will be reported to the Clinical Research Center and the Ethics Committee yearly.

Blood samples and data will be preserved for at least 5 years. All researchers involved in this trial will undertake adequate training before starting their work, and they are required to record detailed information and data truthfully.

The researchers will try their best to make all participants understand the purpose, benefits, and risks of the trial and provide a full and detailed explanation of the trial. If the participants have concerns, they can take the informed consent form home and consider it carefully or discuss it with their family members. The researchers will answer all the questions from the participants to ensure that they are fully informed about the entire trial.

The researchers of each center will discuss the protocol modifications, and the changes will be reviewed and approved by the Ethics Committees before implementation.

The participants will also be required to report their daily meals at the time points stated above. They will be requested to consume meals alone. When reporting, the net weight of food will be calculated using a kitchen scale, and the food will be photographed before and after eating with descriptions of the meal. If the food has a package, a weight and energy ratio will be also photographed. If the quality of the photos is poor or any information is absent, researchers will call back in a timely manner.

Concomitant medications prohibited during the study include glucocorticoid and other hyperglycemic agents.

### Statistical analysis

This study will analyze both the full analysis (FAS) set and per-protocol set and accept the non-inferiority hypothesis only if it is confirmed in both samples. Multiple imputations will be used to handle missing data in the FAS. To avoid potential bias in the results, which may be due to methodological decisions or our datasets, we will perform a sensitivity analysis. Subgroup analyses will be performed according to HbA1c levels, medication history, and diabetes duration. Statistical analysis will be performed using SPSS Ver.21.0 software. Data are presented as mean ± standard error or median (25th–75th percentiles) for continuous variables and as percentages (%) for categorical variables. Baseline and endpoint differences between groups will be analyzed using the independent *t*-test or Mann–Whitney *U* test for continuous variables and the chi-square test for categorical variables. Comparisons within each group (before and after the 3-month intervention) will be performed using a paired *t*-test or Wilcoxon test. Statistical significance will be set at *P* < 0.05. This study will not include interim analyses.

### Dissemination

The results will be disseminated through an international peer-reviewed scientific journal and academic conferences. Standard authorship eligibility guidelines will be followed, and professional writers will not be used.

## Discussion

Antihyperglycemic drugs are currently commonly used for diabetes treatment. However, to date, no trials have compared LCD to antihyperglycemic drugs. Therefore, we will conduct this trial to determine the comparative effects of LCD. Among many antihyperglycemic drugs, sodium-glucose cotransporter 2 inhibitors show similar effects, such as ketogenesis and weight loss, as those of LCD. Among the sodium-glucose cotransporter 2 inhibitors, canagliflozin has the highest capacity for promoting glucose excretion. Therefore, this trial will compare LCD with canagliflozin to evaluate the efficacy of LCD in terms of glycemic control, insulin resistance, and lipid metabolism.

LoCaT is designed as a non-inferiority trial since LCD is more life-altering and patient compliance may not be good. If LCD does not achieve similar effects as canagliflozin, it can be replaced by antihyperglycemic drugs. Therefore, a prerequisite for the clinical application of LCD is that it should have an efficacy similar to that of canagliflozin. Therefore, a non-inferiority study is designed. If the trial results ultimately show that LCD has achieved non-inferiority and may even surpass canagliflozin, we will conduct a superiority analysis.

Although previous studies continuously monitored blood glucose levels after treatment with LCD or sodium-glucose cotransporter 2 inhibitors, only 48-h data were available [13, 14]. Glucose variability is considered an independent driver of increased oxidative stress and inflammation, a predictor of macrovascular and microvascular complications, and an independent risk factor for the prevalence and mortality of cardiovascular events in T2DM [15–17]. In LoCaT, participants will be required to continuously monitor glucose levels by the sensor at the initial and end states of intervention for 14 days so that glucose fluctuation before and after intervention can be observed. Glucose improvement, variability indices, and safety-related indices will be analyzed comprehensively.

Most T2DM patients may simultaneously have several components of metabolic syndrome, including obesity, hypertension, and hyperlipidemia [5]. Participants in this trial will undergo a tolerance test on a given diet at the beginning and end of the intervention, during which changes in insulin and C-peptide will be measured and islet function will be assessed. In addition, plasma lipids, especially fasting and postprandial free fatty acids and triglycerides, will be observed during treatment. LoCaT will also evaluate the body weight and composition and fatty liver disease of all participants, which may be improved after the intervention, and try to find out the intrinsic mechanisms of the effects of LCD on metabolic syndrome compared with those of canagliflozin.

A previous study showed that diabetes combined with hypertension significantly increased all-cause and cardiovascular mortality [18]. In addition, a meta-analysis found that for every 5 mmHg decrease in systolic blood pressure and 2 mmHg decrease in diastolic blood pressure, the risk of stroke in diabetic patients decreased by 13% and 11.5%, respectively [19]. In LoCaT, the blood pressure will be measured monthly to determine its changes, and the mechanism will be preliminarily explored through the assessment of body composition and the renin–angiotensin–aldosterone system.

A meta-analysis reported that both LCD and low-calorie diets decreased hunger in participants with obesity: a low-calorie diet favored increased satiety, whereas LCD favored decreased appetite; appetite suppression was attributed to ketosis (β-hydroxybutyrate ≥ 0.3 mmol/L) [20]. However, the study only evaluated fasting appetite at baseline and after intervention and did not evaluate postprandial appetite. In addition, canagliflozin has ketogenic effects, but there are few reports on its influence on appetite. This present trial will provide new insights into appetite before and after meals in the LCD group. The effects of canagliflozin on appetite will also be explored.

In conclusion, this study aims to determine whether LCD has the same effect as canagliflozin on glucose control in patients with T2DM, and the findings will provide new information on this topic.

## Trial status

The protocol version number is V1.0, dated July 09, 2019. Recruitment began on December 25, 2019. Unfortunately, COVID-19 has interrupted its progress. We expect complete recruitment at all sites in December 2022.

## Supplementary Information

**Additional file 1.** SPIRIT 2013 Checklist: Recommended items to address in a clinical trial protocol and related documents.

## Data Availability

The datasets generated and/or analyzed are available from the corresponding author upon reasonable request.

## Abbreviations

HDL-C: High-density lipoprotein cholesterol
LDL-C: Low-density lipoprotein cholesterol
sd-LDL: Small dense low-density lipoprotein
TC: Total cholesterol
